# Increasing AFM colloidal probe accuracy by optical tweezers

**DOI:** 10.1038/s41598-020-79938-z

**Published:** 2021-01-12

**Authors:** Tomasz Witko, Zbigniew Baster, Zenon Rajfur, Kamila Sofińska, Jakub Barbasz

**Affiliations:** 1 M. Smoluchowski Institute of Physics, Jagiellonian University, Łojasiewicza 11, 30-348 Kraków, Poland; 2 Jerzy Haber Institute of Catalysis and Surface Chemistry, Polish Academy of Sciences, Niezapominajek 8, 30-239 Kraków, Poland

**Keywords:** Techniques and instrumentation, Techniques and instrumentation

## Abstract

A precise determination of the cantilever spring constant is the critical point of all colloidal probe experiments. Existing methods are based on approximations considering only cantilever geometry and do not take into account properties of any object or substance attached to the cantilever. Neglecting the influence of the colloidal sphere on the cantilever characteristics introduces significant uncertainty in a spring constant determination and affects all further considerations. In this work we propose a new method of spring constant calibration for ‘colloidal probe’ type cantilevers based on the direct measurement of force constant. The Optical Tweezers based calibration method will help to increase the accuracy and repeatability of the AFM colloidal probe experiments.

## Introduction

Atomic Force Microscopy (AFM) was first described in 1986 by Binnig et al*.*^1^, where it was used to investigate the topography of hard surfaces. In the following years, many related techniques such as Intermediate Contact, Lateral Forces, Magnetic Force Microscopy were established, including the Colloidal Probe (CP), which allowed the AFM force measurement precision to be transferred to micrometer scale systems.

Parallel to the development of the AFM techniques, intense research on optical trapping and optical tweezers techniques was conducted. From the early 1970s, when the optical trapping technique was introduced by Ashkin^2^, for a long time it was the only method allowing the colloidal particles manipulation in a native environment (i.e. liquid solution) with precisely controlled forces and a high spatial resolution.

The AFM, as well as the Optical Tweezers (OT) allow to investigate protein unfolding^3,4^ cells and microsystems dynamics^5,6^ and interactions in colloidal systems^7,8^. It is surprising that despite the common research field for both techniques, there is only a limited number of papers presenting the simultaneous use of AFM and Optical Tweezers as reference techniques^9,10^. Optical Tweezers employ a highly focused, converged laser beam to trap and manipulate micrometer-size objects, producing forces in the range of piconewtons. In contrast to Atomic Force Microscopy, where force measurements are typically possible only in an axial direction (in selected systems also lateral forces), the Optical Tweezers technique allows to measure interactions in all dimensions^11,12^.

Precise calibration of the AFM cantilever parameters is crucial for all force related experiments. The Colloidal Probe, in contrary to unmodified AFM cantilever, cannot be considered as a simple statically determinable system. Thus, typical calibration methods (Cleveland, thermal noise^13^, Sader^14^, hydrodynamic drag force^15^ and reference cantilevers^16^ methods), used for conventional AFM cantilevers, introduce uncertainties which are difficult to estimate when applied to colloidal probe^17^. For example, using the reference cantilever method, a delicate colloidal particle is exposed to damage in the area being the most relevant for further experiments. Additionally, it is extremely difficult to correctly determine the position of the contact point^18^. The influence of improper positioning of the cantilever was described by Han et al*.*^19^, who showed the physical basis of the induced error (friction forces). This work also presents a method of correct determination of the contact point location. According to Hen, the calibration protocol requires pressing the colloidal particle at least several times. For probing systems in which the colloidal particle is covered with a delicate film (e.g. with adsorbed proteins), it may inevitably lead to the surface damage. Similar problems related to the possibly destructive influence of the friction on the delicate probing layer (present on the surface of a colloidal particle) during the calibration procedure were discussed by Chung et al*.*^20^. The authors proposed the use of electrostatic forces or a thermal noise method as alternative calibration procedures that do not affect the surface of the colloidal probe. Dynamic calibration methods (e.g. thermal noise method) are based on the vibrations with the extremely small amplitude that in some colloidal probe experiments are not comparable to the deflection of the cantilever (nm versus µm).

The colloidal probe type experiments belong to the group of AFM measurements that use the relatively large deflections of the cantilever to probe mechanical properties. Such cantilever deflections are rarely used in measurements with sharp AFM tips. For example, Chen et al.^21^ incorporated the cantilever deflections in the range of 25–30 µm to study micromechanical response of the cell-oocyte complex (COC) matrix. Leporatti et al.^22^ incorporated the deflections of ~ 3.5 µm in the research of the viscoelastic and adhesive properties of lipopolysaccharide-stimulated macrophages. A similar range of cantilever deflections (~ 3.5 µm) is observed in the work of Guz et al.^23^, which intended to answer whether cells (that are highly heterogeneous objects) can be described with parameter like elastic modulus (effective Young’s modulus). To probe mechanical properties of cells, Sokolov et al*.*^24^ indented cells with ~ 2.5 µm cantilever deflections, while Efremov et al*.*^25^ used ~ 3 µm cantilever deflections. It should be emphasized here that such large regime of cantilever deflections is not the only domain of experiments related to the research of cells properties. Bush et al*.*^26^ studied mechanical properties of poly(ethylene glycol) (PEG) hydrogel materials using cantilever deflections of 4–16 µm. Pham and co-workers^27^ used 6 µm cantilever deflections (form − 3 to + 3 µm) in microindentation experiments of soft silicones.

There are many well-described calibration methods for standard AFM cantilevers. However, for colloidal probes the number of calibration methods is limited, and most are either difficult or not very accurate. Beam theory-based methods require very accurate data concerning cantilever geometry^20^. Methods based on finite element method simulations allow to include the influence of the material used to attach the colloidal particle to the cantilever, but this is possible only if its exact distribution on the surface of cantilever is known. In fact, this is possible only approximately. The presented here OT based method of spring constant determination allows to calibrate colloidal probes for experiments requiring large deflections of cantilever (e.g. cell indentation). It is not possible to obtain using other (e.g. the thermal) methods.

Another aspect that poses to be challenging to estimate is the nonlinear behavior of AFM cantilevers that may occur, especially in the case of indentation experiments incorporating relatively large cantilever deflection. Chen with co-workers indicated the presence of nonlinearities for cantilevers, especially for those of small thickness^28^. Moreover, the need to analyze the nonlinearity of the cantilever before starting the experiments was emphasized. The nonlinear behavior of AFM cantilevers has been also confirmed by Ding et al.^29^ in both, finite elements method (FEM) calculations and experiments. Importantly, the authors used commercial AFM cantilevers manufactured by MikroMasch, Inc. The nonlinear behavior of AFM cantilever was also indicated in the work of Mendels and co-workers in a strictly computational work^30^. In another work^31^, the authors focused on investigating the behavior of commercially available AFM silicon cantilevers coated with an Al reflecting layer by FEM. The increase of the thickness of the reflecting layer resulted in the increase of nonlinearities for both, normal and torsional strains. The presented work is especially important in respect to the homemade colloidal probe cantilevers that are partially covered with a relatively thick layer of glue. Such additional layer, similarly to the breakout layer described above, is the reason of the nonlinear behavior of colloidal probe cantilevers.

**Figure 1:**
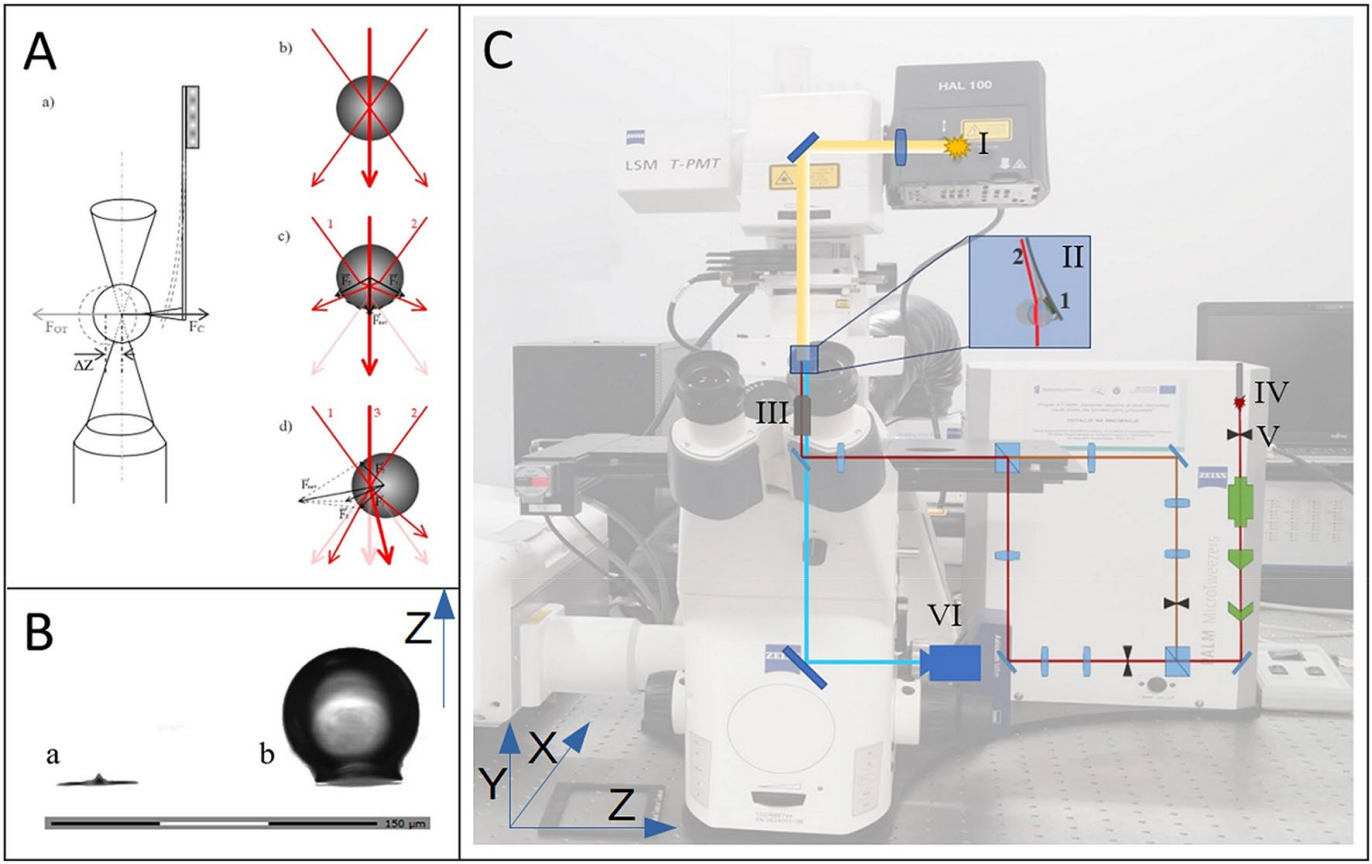
Left panel: (**A**) The scheme of experimental setup (a) and force distribution on a glass particle treated with the laser beam generated by OT (b–d)^32^; (**B**) Transmitted light microscopy image presenting AFM cantilever before (a) and after (b) glass particle attachment.. Right panel: (**C**) The image of experimental setup with the scheme showing the course of the OT laser beam and the optical path of the microscope. I—bright field illumination, II—sample, II.1—AFM cantilever with colloidal probe II.2—OT laser beam, III—trapping objective, IV—OT laser source, V—OT main shutter, VI—camera.

Considering simple physical relations and combining the AFM and OT techniques, we present here a simple, innovative, and precise method of colloidal probe cantilevers calibration. In the described experimental setup (see a in Fig. 1A) the colloidal particle acts as a converging lens for a laser beam (b in Fig. 1A), causing the change in direction of its propagation. Based on the wave-particle dualism, together with its propagation, the photons’ momentum is changing and is transferred onto the particle.

In this manuscript, the colloidal probe calibration method using Optical Tweezers is described. This paper is divided into three main parts. The ‘material and methods’ section is focused on the description of colloidal probes preparation procedure, Optical Tweezers measurements including the calibration of OT stiffness, details of confocal microscopy imaging and the determination of sphere center based on 2D image. In the section “Theory/calculations”, the method of calculation value of the force exerted by OT on a glass bead is described. The point-by-point calibration procedure for determining the spring constant of a colloidal probe is provided in section “Experimental procedure for calibration colloidal probes with optical tweezers”. The sections “Results”, “Discussion” and “Conclusions” provide more details regarding the presented calibration method.

## Materials and methods

### Colloidal probes preparation

Colloidal probes were prepared using NT-MDT Slover Bio atomic force microscope (scanning head SMENA SFC050L). To prepare colloidal probe cantilevers, the following AFM probes were used: CSG10 series (NT-MDT) with a rectangular cantilever and PNP-TR-TL-SPL (NanoWorld) with a triangular (V-shape) cantilevers. Glass spheres with well-defined radius were attached to AFM cantilevers using an epoxy resin by approaching/retracting the AFM probe: (a) the glue, (b) the glass sphere. The image of cantilever before and after sticking the glass sphere is presented in Fig. 1B.

### Optical tweezers measurements

Optical tweezers technique is based on electromagnetic interaction between the laser beam and the trapped diamagnetic particle^33^. There are several physical theories describing this phenomenon. The application of specific theory depends on the ratio between the bead size and the laser wavelength^34^. In our experiment we are using relatively large beads. At this regime of bead sizes/laser wavelength, the ray-optics theory describing the interactions is the most accurate^34^. In the further description we will neglect the influence of the light reflected from the bead surface since it is much smaller than trapping forces^35^. A particle acts as a thick converging lens. A laser beam which enters a bead is refracted towards the center of the sphere. The direction of the beam propagation changes and it affects the momentum. The momentum induces force directing the bead to the center of the trap^36^ (Fig. 1A).

Optical Tweezers experiments were performed using Zeiss PALM MicroTweezers set on Zeiss Axio Observer.Z1 (Fig. 1C) with PALMRobo version V 4.6.0.4 software. OT manipulations were conducted using oil immersion Zeiss EC-Plan Neofluar 100 × 1.3 NA objective (Fig. 1C.III) in air (~ 0.85 NA) and 1064 nm laser (Fig. 1C.IV). To determine the force constant of colloidal probe cantilever, the OT beam was parked at the distant edge of the glued bead and the displacement of the probe from the initial position was measured (with laser trap turned off). After each measurement step the laser power was increased, and the trap was moved to retain the position relative to the bead.

#### The calibration of the OT stiffness

To properly calibrate the AFM cantilever, the precise determination of the force the OT interacts with the glass bead is crucial. In the case of experimental setup without QPD detector the Stokes drag force-based method is the most accurate^32^. The procedure for OT calibration used to determine the optical trap stiffness as a function of laser power has been described in detail by Williams^37^. A diagram showing the configuration of OT calibration in our setup is presented in Supplementary Materials. The trapped bead is accelerated into a known velocity *v* by movement of the microscope stage. Based on Stokes law with implemented Faxen’s correction due to small distance *h* between the sphere center and the surface, it is possible to calculate exerted force *F*_*Z*_ as given^38^:

$${F}_{Z}=\frac{6\pi \mu vr}{1-\frac{9}{16}\left(\frac{r}{h}\right)+\frac{1}{8}{\left(\frac{r}{h}\right)}^{3}+\frac{45}{256}{\left(\frac{r}{h}\right)}^{4}-\frac{1}{16}{\left(\frac{r}{h}\right)}^{5}}$$

where *η* is the media viscosity, and *r* represents the radius of the bead. This allows to calculate the force–displacement dependence and the stiffness constant of the trap. Leach et al.^39^ compared the experimentally determined changes in drag coefficients with the calculated Faxén’s correction and showed a high agreement of the proposed correction with the experimental results. In this work, the authors also took into account the inaccuracy resulting from both, errors in optical methods for determining the particle position, and the surface roughness. And as they show, they are not significant for the obtained results, e.g. 10% increase in drag coefficient occurs at a distance of five radii.

**Figure 2:**
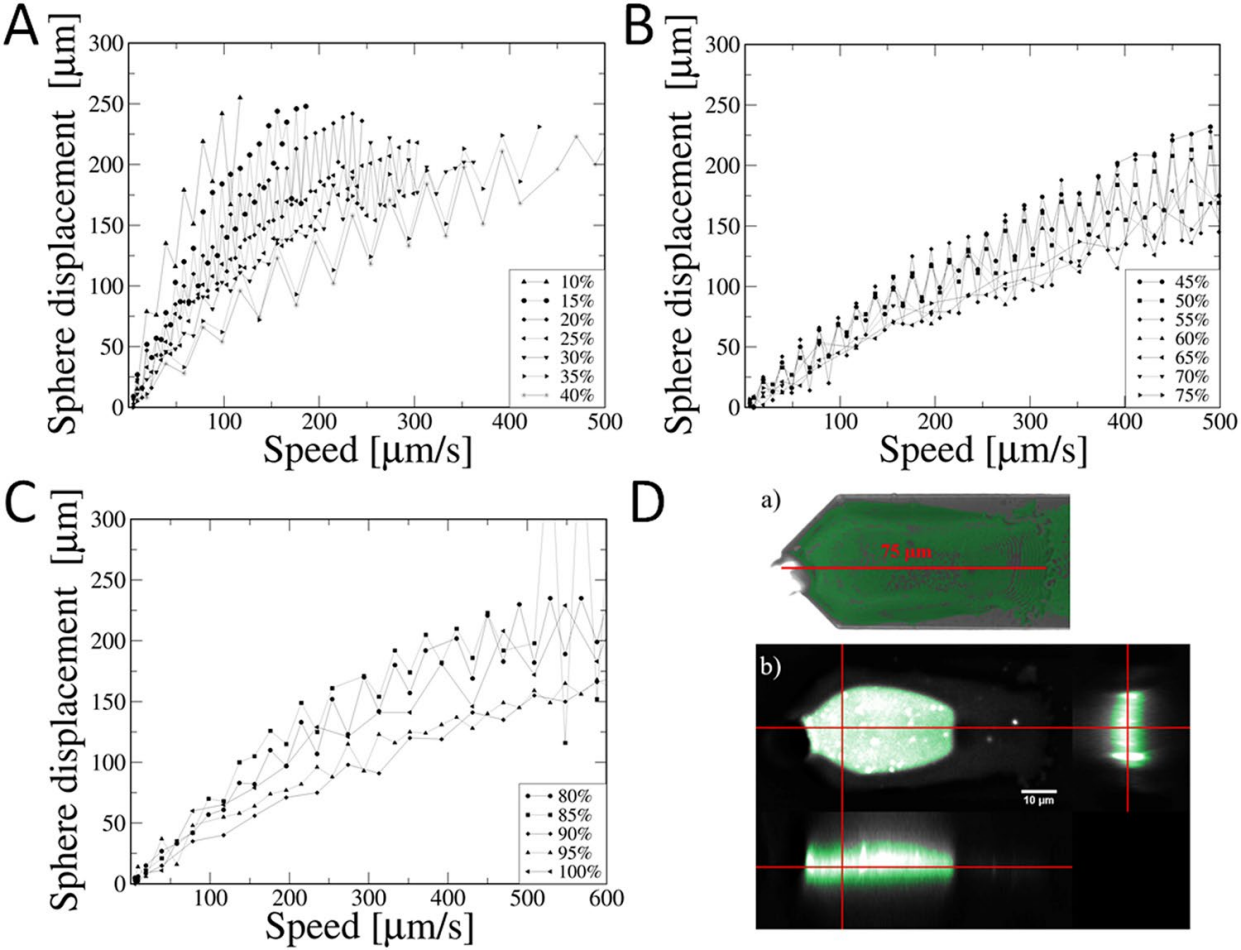
Panels A–C: Calibration curves for determination of the trap stiffness presented as a dependence of the laser beam speed on the sphere displacement. The laser beam power was in the range of: (**A**) 10–40%, (**B**) 45–75%, and (**C**) 80–100%. Panel D: The reconstruction of the epoxy glue distribution on the cantilever: (a) fluorescence microscopy image, and (b) three-dimensional (3D) reconstruction. 3D reconstructions were assembled with a fluorescence microscopy to visualize the distribution of adhesive layers on the cantilever surface. The adhesive layer was marked with a green color. Images were captured on the confocal setup shown in Fig. 1C and then deconvolved using the DeconvolutionLab software^40–42^.

As a result of the procedure described above, we received several calibration curves (Fig. 2A–C) presenting dependences between the glass bead displacement and the speed of the laser beam movement.

The next step of the calibration method was to determine the stiffness of the trap for a specific laser power. During the calibration process, the stage is moving in one direction during one step, and in the opposite direction during the next step^43^. The variable motion of the table is a significant reason for the observed sawtooth waveform (Fig. 2A–C). Another reason for the observed oscillations is the non-zero width of the trap. The particle jumps from one edge of the trap to the other. It is important to define the power range in which the rigidity of the trap changes linearly with increasing power. This allows to determine the equation describing the trap and thus accurately estimate the force magnitude the trap interacts with the object during the experiment. In the configuration used in our experiments, the maximum laser power (100%) corresponds to 5 W of electrical power.

The error in determining the trap stiffness is transferred directly to the accuracy of determination of the force constant. For each trap, the uncertainty of trap stiffness determination is clearly defined in the calibration procedure. To determine the trap stiffness, linear regression for the linear range of the trap operation is applied.

Except the viscous drag force method that we incorporated to calibrate OT, several other calibration methods can be used: sequential impulse response^44^, the equipartition theorem method^45^, power spectrum analysis^46^, method based on an autoregressive model^47^, calibration with differential interference contrast signals^48^, calibration method in the presence of motion blur^49^ and other. A fine comparison of the calibration methods was performed using 1 µm diameter polystyrene beads by Capitanio et al.^48^. The viscous drag force calibration method appeared to have the smallest typical error of 2%.

### Confocal microscopy imaging

Confocal microscopy imaging was performed using Zeiss LSM 710 set on Zeiss Axio Observer.Z1 (Fig. 1C) with ZEN black version 8.1.0.484 software and no immersion Zeiss NA EC-Plan Neofluar 20 × 0.5 objective.

The amounts of glue cannot be fully controlled in the process of glass sphere attachment to AFM cantilever. To obtain fluorescence image of the epoxy glue distribution (Fig. 2D), it was mixed with fluorescein. Fluorophore was excited with 488 nm laser. A transmitted-light image was collected simultaneously with a fluorescence image. A confocal image was analyzed using Fiji software^50^. For a PSF creation we applied the PSF Generator software^51,52^ using Born & Wolf 3D Optical Model^53,54^ with parameters matching the collected image with best computation accuracy. The deconvolution of the fluorescence image was performed in the DeconvolutionLab software^55^ using the Richardson-Lucy algorithm^40,41^ with 10 iterations.

### The determination of sphere center based on 2D image (software automated)

Relying on the fact that optical microscope used in this experiment provides high contrast and resolution, surface of the sphere can be easily visualized and binarized using appropriate threshold function in dedicated software. The determination of the exact sphere curvature course, followed by basic geometrical relationships application, allows to directly establish the sphere center. Because the experimental setup ensures that the particle is fixed in X axis (microscope Z-axis) the only possible movement in the system may occur in Z and Y axes. The determination of sphere center displacement in two-dimensional system comes to calculating the change in coordinates of a circle contained in focus plane of the microscope.

**Figure 3:**
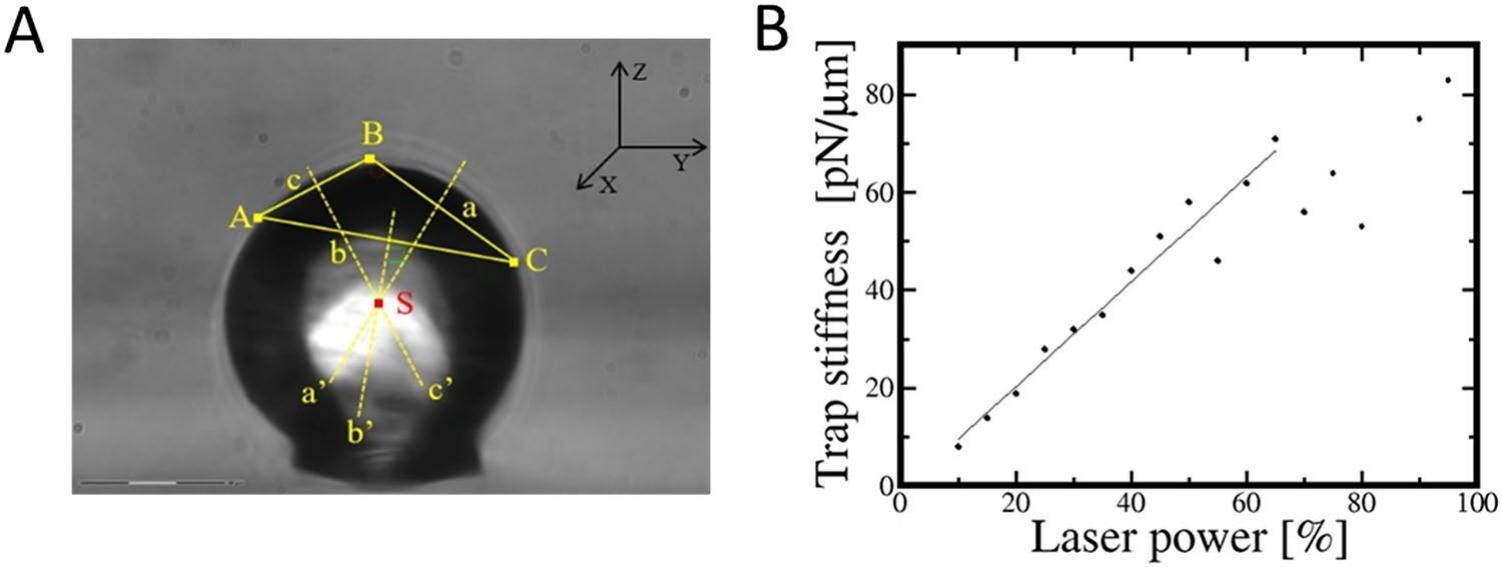
(**A**) The determination of sphere center based on 2D image (analytical approach); (**B**) The dependence of a trap stiffness on the laser power.

To designate the center of a circle, three points forming a triangle at its edge need to be drawn (A, B, C). The intersection of normal vectors (a’, b’, c’) to the triangle sides (a, b, c) defines the center of the circle (S) (Fig. 3A). Alternatively, for non-ideally spherical particles, the mean value of the coordinates of multiple points randomly selected around the periphery of the particle can be used.

Because the whole process is not mathematically intricating, it may be easily automated using the ImageJ^56^ or other image processing dedicated software macro. This method allows simultaneous deflection tracking in Z and Y-axes.

The diameter of the particle attached to the cantilever in a described optical configuration is about 500–1500 pixels (for the nominal particle diameter of 15–45 µm). The error in determining the location of the particle center by 1 pixel introduces the uncertainty of the force constant of about 0.07–0.2%. Assuming a single measurement, the error of 5 pixels gives less than 1% uncertainty in determination of the particle center.

## Theory/calculations

As mentioned earlier, the Optical Tweezers technique is based on a laser beam deflection in a dielectric particle. There are several theories describing this phenomenon depending on a particle diameter to laser wavelength ratio^42^. For the ratio higher than 10, a geometric optics approach is the most accurate (Fig. 1A.b–d)^57^. The setup used for calibration experiments (Fig. 1C) employs 1064 nm laser and glass beads of at least 30 µm in diameter.

As described in papers^58,59^, forces *F*_*Z*_ (parallel to the beam propagation vector) and *F*_*X*_ (orthogonal to the beam propagation vector) exerted on spherical objects are linearly correlated with the laser power *P*:

$${F}_{Z}=\frac{nP}{c}\left\{R\left.\, sin2\theta -\frac{{T}^{2}\left[\mathrm{sin}\left(2\theta -2r\right)+Rsin2\theta \right]}{1+{R}^{2}+2Rcos2r}\right\}\right.$$

$${F}_{X}=\frac{nP}{c}\left\{1+R\left.\, cos2\theta -\frac{{T}^{2}\left[\mathrm{cos}\left(2\theta -2r\right)+Rcos2\theta \right]}{1+{R}^{2}+2Rcos2r}\right\}\right.$$

where *n* is a medium refraction index, *c* is a speed of light, *T* and *R* are the Fresnel transmission and reflection coefficients and *θ* and *r* are the and refraction and incidence angles.

## Experimental procedure for calibration colloidal probes with optical tweezers

The AFM colloidal probe calibration should be conducted as follows:

1. Align the glass bead at microscope and Optical Tweezers focal planes. The surface of the colloidal probe cantilever should be oriented perpendicular to the force exerted by the optical tweezers. In the case of inaccurate orientation of colloidal probe in respect to the force generated by OT, the value of the force needs to be corrected by multiplying it by the cosine of the angle between the applied force and the normal to the cantilever surface.
2. Calculate the bead center position with OT laser turned off, as its initial position.
3. Set a known power for OT laser.
4. Place the OT beam at the distant edge of the bead. The laser beam can be positioned anywhere but not at the center of the bead. As the laser is focused on the bead, part of a primary beam can be obscured by the cantilever. To avoid such a situation, the laser should be set as far as possible from the particle center.
5. Calculate the bead center position and its displacement.
6. Increase the laser power. The colloidal probe should move slightly towards the beam as it experiences a higher force.
7. Move the beam to retain its position at the edge.
8. Repeat the procedure more than 5 times.

## Results

The calculated stiffness of the trap corresponding to the specified laser power is presented in Fig. 3B. The range in which the trap preserves the linear characteristics has been distinguished on the graph. The points being beyond this range (over 60% of laser power) are not considered in further calculations. By matching a straight line to a graph, a function combining trap stiffness (*St*) with the laser power (*P*) set in the software can be determined: *St* = *1.039P* + *0.2582*. This allows to calculate the optical trap stiffness for a specified laser power.

**Figure 4:**
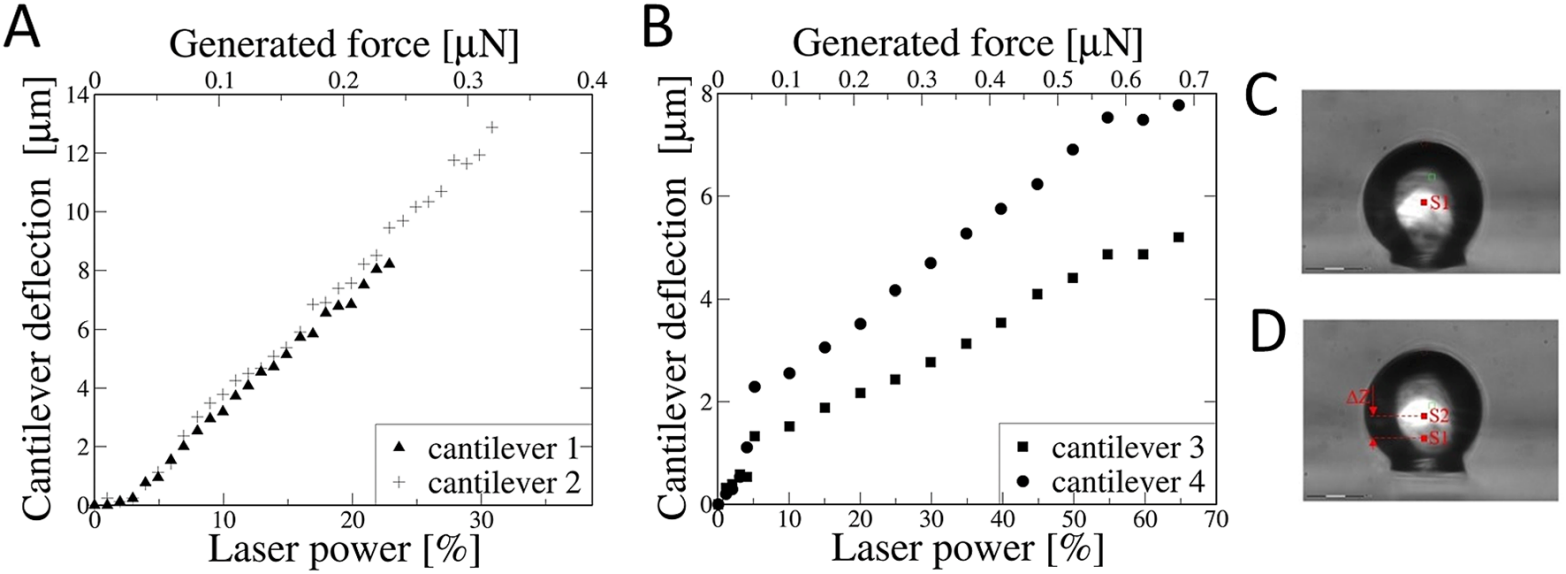
Cantilever deflection under the influence of increasing OT generated force for (**A**) rectangular cantilever (CSG10, NT-MDT) and (**B**) V-shape cantilever (PNP-TR-TL-SPL, NanoWorld). All cantilevers have been modified to colloidal probes. Particle position at lowest (**C**) and highest (**D**) applied laser power.

In the proposed calibration method, the displacement of optical center of a colloidal particle as a result of interaction with the OT laser beam has been determined (Fig. 4). Force generated by the OT is shifting particle in *Z* direction (Fig. 4C,D). Direct observation of the experiment is presented in supplementary video 1 and video 2. The position of particle is a derivative of the applied optical force. According to the first Newton Dynamic Law this force is equal to the force coming from cantilever elasticity (spring constant) which results in a stable position of the glass sphere.

**Figure 5:**
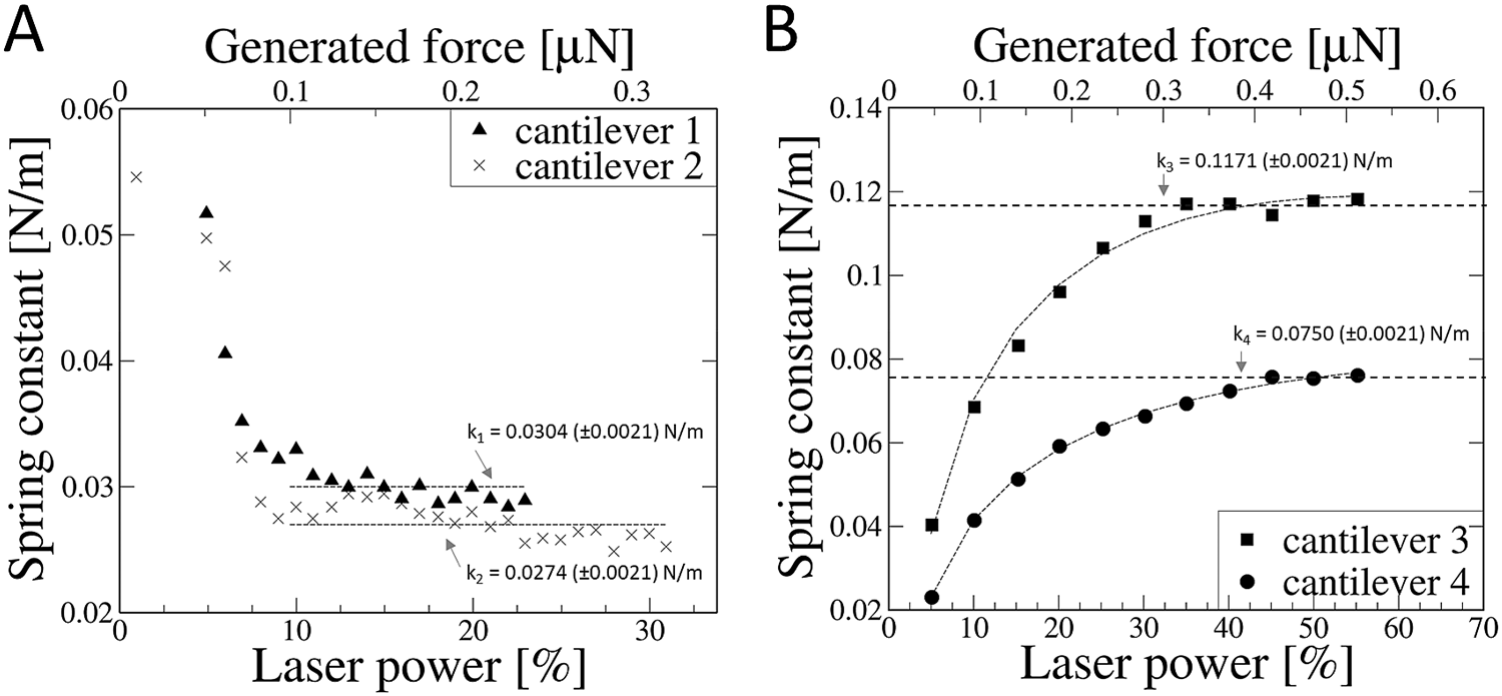
The dependence of the force constant (k) on the OT laser power/generated force for A: rectangular cantilevers (CSG10, NT-MDT) modified to colloidal probes, and B: triangular (V-shape) cantilevers (PNP-TR-TL-SPL, NanoWorld) modified to colloidal probes.

The spring constants (*k*) of individual colloidal probe for each subsequent value of cantilever deflection can be determined using the Hooke’s law relationship: *F* = *k* Δ*Z*, where *F* represents the force influencing the colloidal probe, *k* is the spring constant of cantilever and Δ*Z* is the sphere center displacement. The spring constant/laser power dependence graphs (Fig. 5) enable to determine the OT generated force range in which the spring constant (k) is represented by fixed value. The elasticity constant of rectangular cantilevers modified to colloidal probes does not change over a wide range of the applied OT laser power (Fig. 5A). In the case of triangular (V-shape) cantilevers (Fig. 5B), the logarithmic function was fitted. Since it is impossible to define the exact equation describing the behavior of the V-shaped cantilever without fully knowing the distribution of the glue on the cantilever surface, we propose a logarithmic fit which combines simplicity and high accuracy in the described range. This function binds laser power (*P*) to the spring constant of cantilever (*k*). The spring constant of V-shape cantilevers cannot be described by a fixed value for the entire operating range, the spring constant appears to be represented by a fixed value only in a narrow range of action. Functions fitted to the spring constant/laser power dependence obtained for V-shape cantilevers are as follows:

$${k}_{3}\left(P\right)=\left\{\begin{array}{l}P:0.0528\mathrm{ln}\left(P\right)-0.0009P-0.0422 \\ P>30\%: 0.1171 \pm 0.0021\end{array}\right.$$

$${k}_{4}\left(P\right)=\left\{\begin{array}{l}P:0.0289\mathrm{ln}\left(P\right)-0.0003P-0.0221 \\ P>45\%: 0.0750 \pm 0.0021\end{array}\right.$$

The elasticity constants for all studied here AFM cantilevers obtained in the experiment agree on the order of magnitude with the manufacturer's declared values. The results are summarized in Table 1.

**Table 1:** Juxtaposition of parameters provided by the manufacturer with the results obtained in the experiment.

Probe no	Cantilever type	*k* declared by manufacturer (N/m)			*k* experimental (N/m)^a^	Function fitted^b^
		Min	Typical	Max		
1	CSG10	0.01	0.11	0.5	0.0304	–
2					0.0274	–
3	PNP-TR-TL-SPL	0.02	0.08	0.11	0.1171 for $P>30\%$	*k*_*3*_= {$P:0.0528\mathrm{ln}\left(P\right)-0.0009P-0.0422$
4					0.0750 for $P>45$	*k*_*4*_= {$P:0.0289\mathrm{ln}\left(P\right)-0.0003P-0.022$

^a^The average values are derived from the measuring points of the linear cantilever operation region (Fig. 5).

^b^In the case of cantilevers 3 and 4, the logarithmic function describing characteristics of the cantilever is fitted to experimental points (Fig. 5B).

## Discussion

The proposed here calibration procedure is fast, universal and can be applied for cantilevers of unrestricted geometry. The approach described in this paper implements the direct force constant measurement, which helps to overcome several assumptions and approximations present in several commonly used calibration methods. The cantilever calibration based on OT is uninfluenced by numerous probe preparation imperfections such as the lack of symmetrical placement of a colloidal particle or imprecise fabrication of the cantilever itself. Furthermore, mechanical properties of glue (i.e. Young’s Modulus) and its distribution on the probe are usually undefined, but severely influencing the characteristics of the system. Even the location of the tip on the cantilever (the distance of the tip from the cantilever end) is known to affect the cantilever stiffness^60^. The imprecise attachment of colloidal particle introduces significant contribution to the cantilever stiffness because of the particle mass. The OT based calibration method allows to determine the spring constant without the need of modeling the mentioned factors. In the case of homemade colloidal probes, the amount of glue immobilizing the particle may be comparable with the mass of attached particle itself. The lack of knowledge about its mass, quantity and distribution on the cantilever introduces considerable uncertainty while employing existing calibration methods (i.e. based on the resonance frequency modeling^61^).

The results presented here show that cantilevers with more complex geometry (i.e. V-shape) display nonlinear response characteristics in wide operation range (Fig. 5B). In fact, the spring constant (*k*) can only be recognized as fixed value in a very narrow range of operation. The nonlinear characteristics of the triangular AFM cantilevers has been described earlier by Korayem et al.^60^. Computer generated virtual cantilever models confirm the behavior of V-shape probes observed here. The nonlinear behavior of AFM cantilevers has been confirmed also using the finite element method (FEM) simulations in several articles^28–31^. To support our experimental findings related to nonlinearity of AFM cantilevers, we provide the FEM simulations in Supporting Materials. In fact, the spring constant of V-shape cantilevers should be considered as a force function. For a specific value of force during the force-spectroscopy experiment, the appropriate value of spring constant should be determined based on the function derived within the calibration process. It is the only way to improve the quality of the force-spectroscopy results and make them more comparable between various research groups, which seemed to be one of the biggest struggles in this field so far^62^.

The precise spring constant determination helps to avoid significant errors in all force-related colloidal probe experiments. The direct approach presented in this paper not only allows to determine the spring constant avoiding the influence of factors listed above, but it also provides a precise information about the characteristics of cantilever deflection as a function of increasing force generated by OT laser beam. Such knowledge allows to establish to what extent the deformation is linear and thus the spring constant can be recognized as fixed value in the case of rectangular cantilevers. For V-shape cantilevers, it derives the spring constant/force formula to calculate exact spring constant for the specific force applied in the experiment.

## Conclusions

The exact spring constant determination of AFM cantilever is essential due to the influence on the precision and efficiency of force-related AFM measurements. The calibration of colloidal probes is exceptionally difficult. In the case of presence of the colloidal particle displaying a large mass concentrated at the end of the cantilever, the resonance-based calibration methods do not produce the sufficient accuracy. The presented here calibration method minimizes any uncertainty associated with the geometry of the colloidal probe type cantilever and allows a significant increase in the accuracy of the results. In addition, it makes the calibration process quick, convenient, and highly repeatable.

There are several works describing the use of colloidal probes in the regime of large cantilever deflections during experiments^63–65^. The OT based calibration method allows to obtain more accurate results and to work in an extended regime of applied forces/cantilever deflection values in comparison to other methods. Therefore, this method is especially useful in biophysical research concerning AFM indentation.

The universal character of this method allows to calibrate, using OT, homemade colloidal probe cantilevers and also factory made pre-calibrated colloidal probes, especially in the case of uncertainty of the used calibration method accuracy (when single *k*-constant is provided). In the case of cantilevers with V-shape geometry, the assumption of their linear characteristic is a simplification and they cannot be described by a single spring constant. The additional calibration process and estimating spring constant/force formula, especially for V-shape probes will allow to use them in the wide range of applied forces.

Kim et al.^66^ compared several methods of cantilever calibration, but none of them allows to determine the force constant as a function of the applied force (deflection), which is essential for homemade cantilevers and experiments involving relatively large cantilever deflection. The OT based calibration method allows to obtain accuracy comparable to the static force calibration method described by Kim et al*.* (2%)^66^. However, the OT based calibration method is less complex and easy to apply.

The cantilever calibration method presented in this paper is fully applicable in routine AFM experiments and requires only a stable mounting of the AFM cantilever in the OT optical path. The high accuracy of the object positioning in the OT allows a precise determination of the cantilever spring constant.

## Supplementary Information

Supplementary Information.

Supplementary Video 1.

Supplementary Video 2.
