# Supplementary material for: Increasing AFM colloidal probe accuracy by optical tweezers
Source: Sci Rep. 2021 Jan 12;11:509. doi: 10.1038/s41598-020-79938-z (PMC7804458; doi:10.1038/s41598-020-79938-z)
Supplement: Supplementary file 1 — Supplementary Information. [file 41598_2020_79938_MOESM1_ESM.docx]

**Increasing AFM colloidal probe accuracy by Optical Tweezers**

**Tomasz Witko^1,2^, Zbigniew Baster^1^, Zenon Rajfur^1^, Kamila Sofińska*^1,2^ & Jakub Barbasz*^2^**

1 - M. Smoluchowski Institute of Physics, Jagiellonian University, 30-348 Kraków, Łojasiewicza 5 11, Poland.

2 - Jerzy Haber Institute of Catalysis and Surface Chemistry, Polish Academy of Sciences, Niezapominajek 8, 30-239, Kraków, Poland.

*e-mail: ncbarbas@cyf-kr.edu.pl (J.B), kamila.sofinska@uj.edu.pl (K.S.)

Direct observation of the experiment is presented in supplementary video: 01.avi, 02.avi

**The laser stability**

The stability of the laser was confirmed experimentally by a triple calibration performed for five additionally prepared colloidal probe cantilevers. For the rectangular cantilever, R^2^ of the linear fit is presented below for subsequent measurements (laser power range 0-60%).

a) cantilever N1, geometry rectangular R_1_^2^=99,59, R_2_^2^=99,49, R_1_^2^=99,52

b) cantilever N2, geometry rectangular R_1_^2^=99,59, R_2_^2^=99,54, R_1_^2^=99,61

c) cantilever N3, geometry V-shape

d) cantilever N4, geometry V-shape

e) cantilever N5, geometry V-shape

**Figure S1**. (a, b) - calibration curves for rectangular cantilevers, (c-e) calibration curves of v-shaped cantilevers

The significant influence of the parameter *h* on the accuracy is observed only for *h* > *r*. In the experimental setup described in the Materials and Methods section, the inverse relationship is observed. The *h* value is directly determined based on the displacement of microscope objective as a difference between the glass surface and the imaging plane.

In example: in the case of misestimation of *r* / *h* of 10% which for beads with 50 µm in diameter, it would be 2,5 µm. In reality, such misestimation for currently available semi-automated microscopes is rather impossible. In such a case, the error in estimation the force would be smaller than 2%.


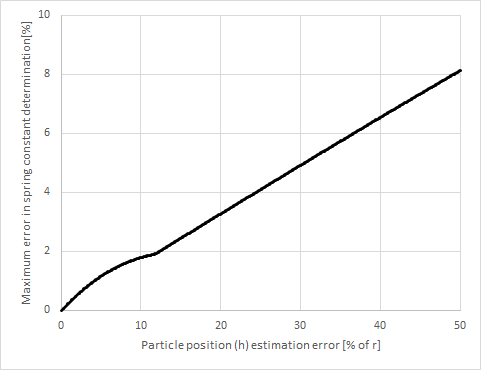


**Figure S2**. The impact of the estimation error of distance between particle center and surface (h) on error of spring constant.

The relation between bread position error in Z direction and error of the spring constant estimation. Calculations based on Faxen’s correction equation.

| Cantilever type | OT method  [N/m] | Sader  [N/m] | estimation from cantilever geometry [N/m] | Manufacturer datasheet  [N/m] |
| --- | --- | --- | --- | --- |
| CSG01 | 0,150953877467642 | 0.0343 | 0,00312 | 0.003-0.13 |
| CSG01 | 0,131888381986942 | 0.0433 | 0,00502 | 0.003-0.13 |
| MLCT cantilever C | 0.0688412512969656  (for small deflection)  0,110297557168269  (for large deflection) | 0.0222 | 0,00061 | 0.01-0.02 |
| MLCT cantilever C | 0.0341926788103005  (for small deflection)  0,096160718678269 (for large deflection) | 0.0284 | 0,00067 | 0.01-0.02 |
| MLCT cantilever C | 0.0296068991686012  (for small deflection)  0,083950599919953  (for large deflection) | 0.0295 | 0,00035 | 0.01-0.02 |

Table S1

Comparison of the two calibration methods (Sader and estimation from cantilever geometry) with the manufacturer's data and the results obtained with the OT method.


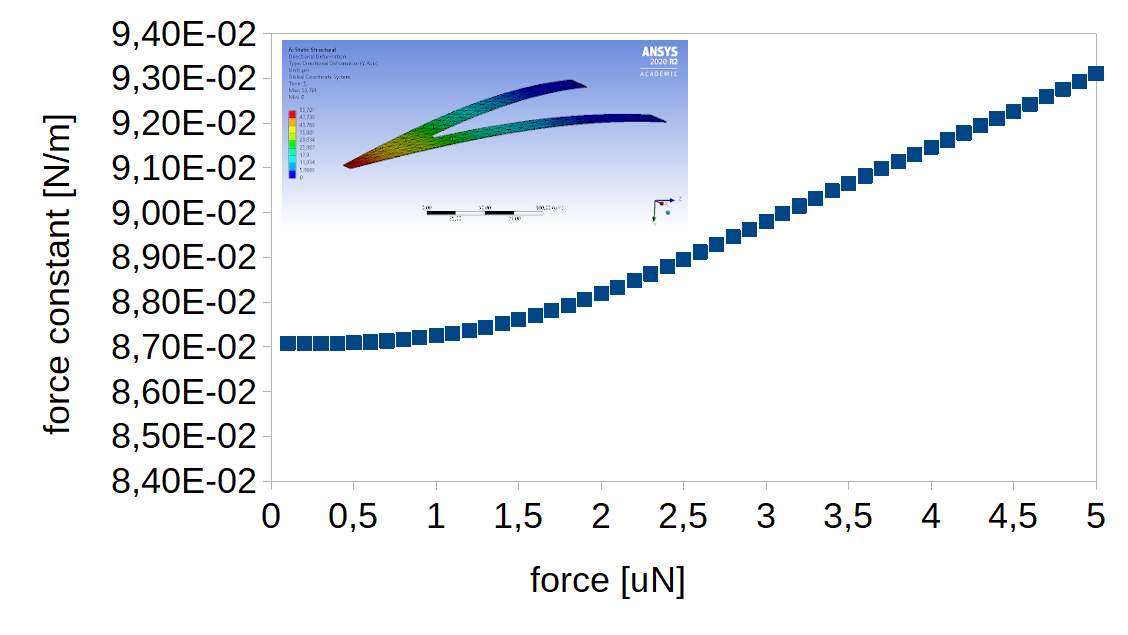


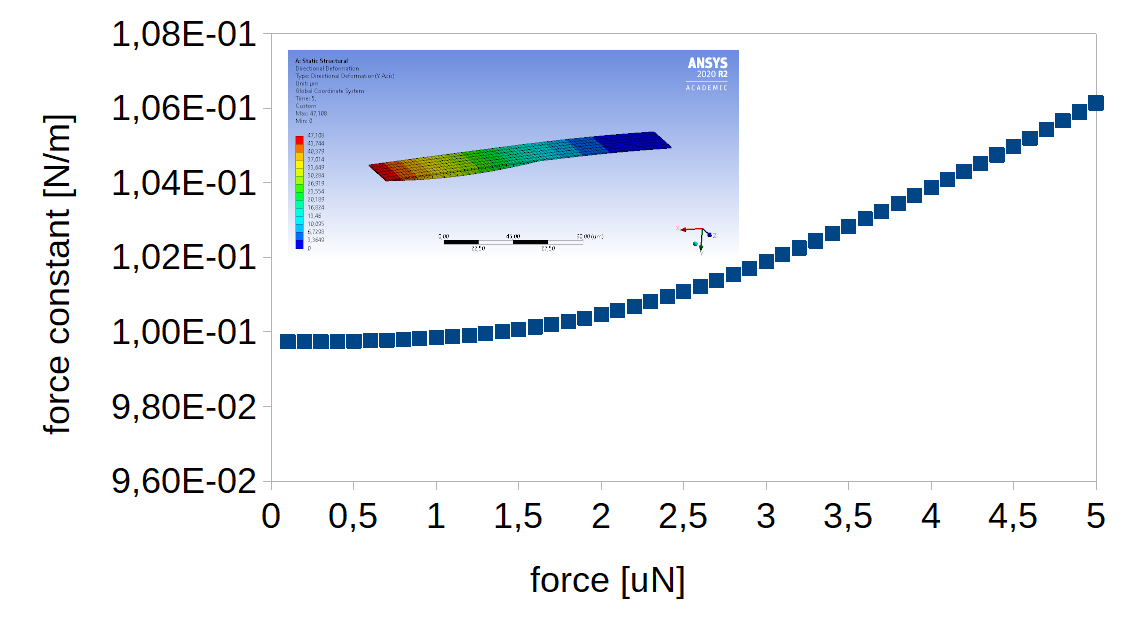


The graphs show the nonlinearity of force constant in simple models of two cantilevers calculated by the finite element method using ANSYS software. Correspondingly, the first one (upper panel) presents results for a V-shaped silicon cantilever, and the second graph (lower panel) shows the results for a rectangular cantilever half covered with epoxy resin, which is typically used for sticking glass beads to prepare the colloidal probe type cantilevers.


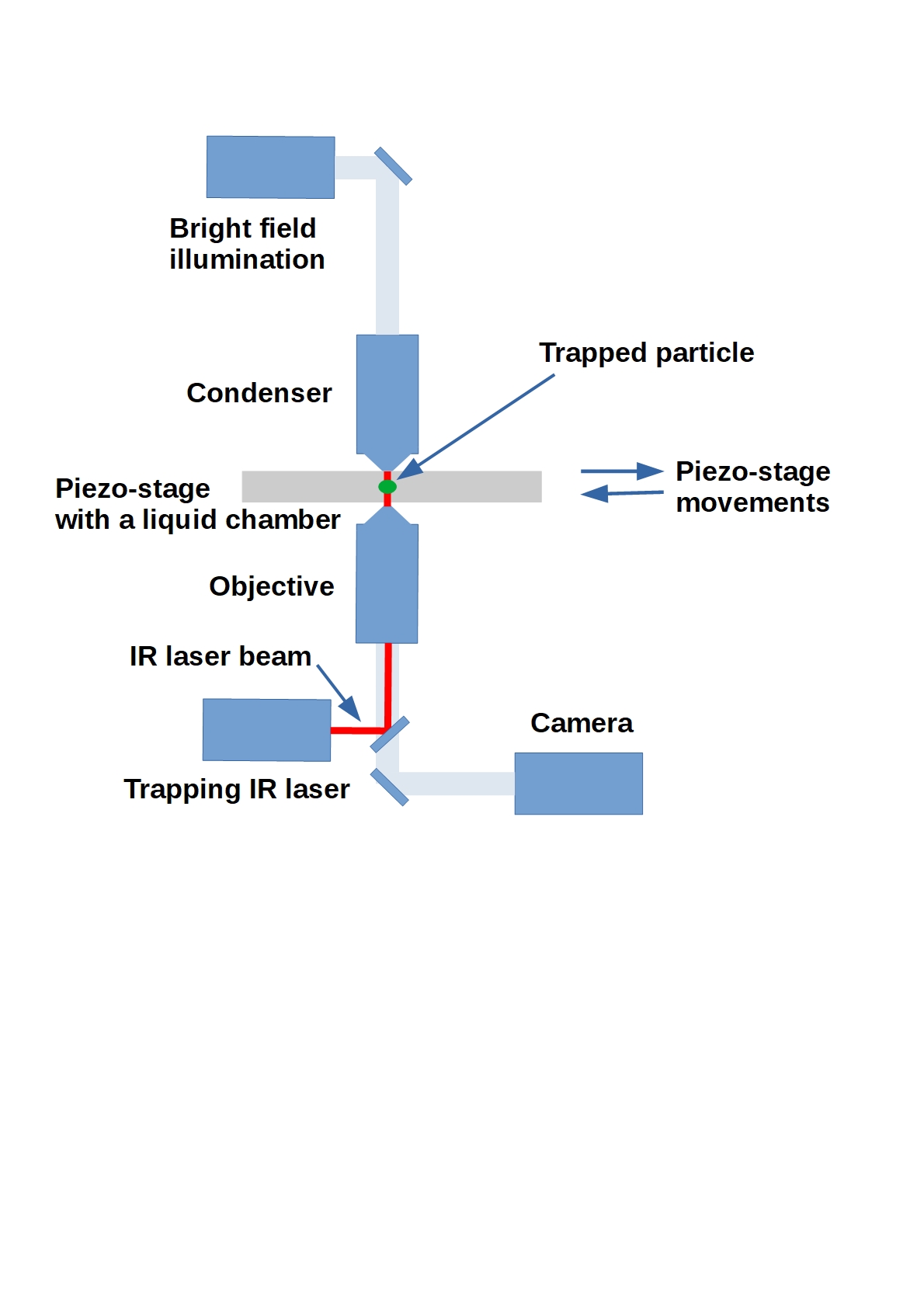


A diagram showing the configuration in which the calibration of the optical tweezers was performed.
